# N-Alkylpyridinium sulfonates for retention time indexing in reversed-phase-liquid chromatography-mass spectrometry–based metabolomics

**DOI:** 10.1007/s00216-021-03828-0

**Published:** 2021-12-15

**Authors:** Rainer Stoffel, Michael A. Quilliam, Normand Hardt, Anders Fridstrom, Michael Witting

**Affiliations:** 1 Research Unit Analytical BioGeoChemistry, Helmholtz Zentrum München, Ingolstädter Landstraße 1, 85764 Neuherberg, Germany; 2 National Research Council Canada, Biotoxin Metrology, 1411 Oxford Street, Halifax, N.S B3H 3Z1 Canada; 3 Merck, Frankfurter Straße 250, 64293 Darmstadt, Germany; 4 Metabolomics and Proteomics Core, Helmholtz Zentrum München, Ingolstädter Landstraße 1, 85764 Neuherberg, Germany; 5 Chair of Analytical Food Chemistry, TUM School of Life Sciences, Technical University of Munich, Maximus-von-Imhof-Forum 2, 85354 Freising, Germany

**Keywords:** Metabolomics, Retention time indexing, Reversed-phase, Metabolite annotation

## Abstract

Chromatographic retention time information is valuable, orthogonal information to MS and MS/MS data that can be used in metabolite identification. However, while comparison of MS data between different instruments is possible to a certain degree, retention times (RTs) can vary extensively, even when nominally the same phase system is used. Different factors such as column dead volumes, system extra column volume, and gradient dwell volume can influence absolute retention times. Retention time indexing (RTI), routinely employed in gas chromatography (e.g., Kovats index), allows compensation for deviations in experimental conditions. Different systems have been reported for RTI in liquid chromatography, but none of them have been applied to metabolomics to the same extent as they have with GC. Recently, a more universal RTI system has been reported based on a homologous series of *N*-alkylpyridinium sulfonates (NAPS). These reference standards ionize in both positive and negative ionization modes and are UV-active. We demonstrate the NAPS can be used for retention time indexing in reversed-phase-liquid chromatography-mass spectrometry (RP-LC–MS)–based metabolomics. Having measured >500 metabolite standards and varying flow rate and column dimension, we show that conversion of RT to retention indices (RI) substantially improves comparability of retention information and enables to use of RI for metabolite annotation and identification.

**Supplementary Information:**

The online version contains supplementary material available at 10.1007/s00216-021-03828-0.

**Figure:**
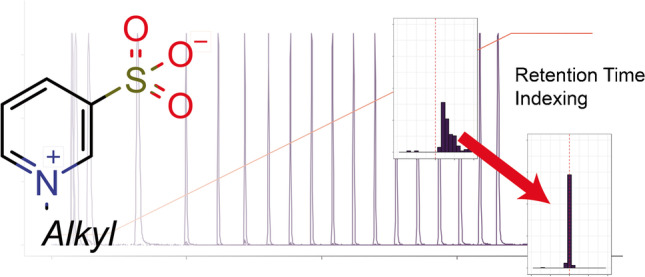
Graphical Abstract

## Introduction

Liquid chromatography-mass spectrometry (LC–MS) is one of the premier methods for the analysis of metabolites. Although powerful, mass spectrometric (MS^1^) analysis alone cannot provide complete identifications due to the existence of several possible isobaric structures for a given molecular weight (if low resolution) and isomeric structures for a given elemental composition (if high resolution). Tandem mass spectrometry (MS^2^) can add an additional layer of information, but several possible candidates might still exist due to similar or identical fragmentation of isomeric species. Orthogonal information such as retention times (RTs) derived from chromatographic separation or collisional cross sections (CCS) from ion mobility experiments are highly suitable to differentiate isomeric structures [1, 2]. The Metabolomics Standard Initiative (MSI) introduced levels of identification for metabolites based on the collected evidence towards particular identifications. The highest level of identification is achieved by comparing an analytical reference standard with those of the measured metabolite feature under identical analytical conditions using a minimum of two independent physicochemical properties [3]. In the case of LC–MS, RT, MS^1^, and MS^2^ using the same chromatographic setup have to match in order to refer to a metabolite as identified. However, it is very unlikely that a single laboratory holds reference standards for all metabolite in-house. Therefore, sharing of information (RT, MS, MS/MS) becomes very important for the wider metabolomics community. Development of novel tools such as RT prediction will enable incorporating RTs at an early stage of metabolite identification, can reduce the number of false-positive assignments and annotations, and helps to speed up the task of metabolite identification, but also requires available training data [4].

Therefore, sharing of RTs for cross-lab comparisons might be useful for enhanced metabolite identification. However, the lack of standardization of chromatographic conditions and the use of different instrumentation complicates the use of retention information. In contrast to mass or CCS, which represent molecular properties, RTs are system properties that arise from the combination of chromatographic equipment, employed mobile and stationary phases, and separation conditions (e.g., flow rate and temperature). Even nominally, the same separation conditions on two different instruments can yield vast differences in absolute RTs due to factors such as column dead volume, system extra column volume, and gradient dwell volume. Even within a single lab, RTs can shift substantially due to deterioration of the column and/or mobile phase or due to different batches (differences in solvent composition, pH adjustment etc.). Therefore, retention information is not used regularly for metabolite identification across different labs. Different approaches to tackle this problem have been developed.

In gas chromatography, RTs are commonly converted to retention indices (RI) by referencing the RT of a given substance to a set of reference standards. This retention time indexing (RTI) is well established and allows the cross-referencing of different separations performed under similar but not exactly the same conditions. The Kovats index, which uses a homologous series of *n*-alkane reference standards spiked into the sample, is the most used RTI system in GC and has also been applied in metabolomics [5, 6].

Different RTI systems have been proposed for LC-based separation, each providing different advantages and disadvantages. Aderjan and Bogusz introduced an RTI system based on a series of 1-nitroalkanes. This substance class shows strong absorbance between 200 and 230 nm. However, they ionize only in negative ionization mode in electrospray MS [7]. Different other substances have been suggested as RTI markers, e.g., alkyl phenones and phenolic esters, reviewed elsewhere [8]. Nitroalkanes and fatty acid amides have been also used for RTI in metabolomics [9]. These RTI systems have also been combined with different in silico analysis methods for tandem MS spectra, CFM-ID. CSI:FingerID, MassFrontier, and MetFrag [10–12]. Based on a set of measured RI values, an artificial neural network was trained to predict RI values for candidates from in silico methods. This combined method was able to improve the average rank of candidate molecular structures [13]. Recently, Zheng et al. established an RTI system for derivatized molecules. 2-Dimethylaminoethylamine (DMED)-labeled fatty acids served as indexing substances for DMED-labeled carboxylic acids. Amine compounds were labeled with 4-(*N*,*N*-dimethylamino)phenyl isothiocyanate (DMAP) and DMAP-labeled fatty amines served as RI standards. Based on their RTI system, the authors compared different chromatographic setups and could show that RI is much more comparable than RT [14]. However, most metabolomic experiments detect metabolites in their native, underivatized state. Therefore, a solution for RTI for unlabeled substances is required. A further disadvantage of the presented substances for RTI is that for positive and negative ionization modes, different substances are used, resulting in two different sets of RI databases. *N*-alkylpyridinium 3-sulfonates (NAPS) have been suggested as promising candidates for the normalization of RT data by conversion to RI [15]. Recently, they have been used for the normalization of LC–MS mycotoxin determination [16]. A recent review by Rigano et al. summarizes historic development and recent advances of RI approaches in LC [17].

We show that NAPS can be used for the normalization of retention information of RP-LC–MS-based metabolomics. In this study, we have measured > 500 metabolite standards of which > 150 showed strong retention under the chosen conditions allowing to construct an RTI database. In order to explore the possibility to normalize for different conditions, we systematically varied the flow rate as well as performing separation in an HPLC instead of UHPLC format. NAPS-based RTI allowed normalization of retention information and enabled the identification of metabolites from *Caenorhabditis elegans* metabolite extracts measured under different analytical conditions.

## Material and methods

### Chemicals

Acetonitrile (MeCN), methanol (MeOH), formic acid (FA), and chloroform (CHCl_3_) were purchased from Sigma-Aldrich and were of LC–MS grade (Sigma-Aldrich, Taufkirchen Germany). Metabolite standards used in this study were derived from the Mass Spectrometry Metabolite Library of Standards (MSMLS) (Sigma-Aldrich). Standards were prepared as indicated in the MSMLS manual. For initial library creation, each row on the plate was pooled yielding a mixture with up to 8 different non-isomeric and non-isobaric metabolites. This yields 52 individual mixtures. For all further experiments, always one plate was pooled. The NAPS RI standards in the form of a reference material (RM-RILC) composed of a mixture of 20 NAPS in solution (100 μM each) were provided by the National Research Council Canada (Halifax, NS, Canada, https://www.nrc-cnrc.gc.ca/eng/solutions/advisory/crm/list_product.html). We refer to this mixture through the text as NAPS.

### Chromatographic methods

#### LC–MS system 1

For the first LC–MS system, a Waters Acquity UPLC (Waters, Eschborn, Germany) was coupled to Bruker maXis UHR-ToF–MS (Bruker Daltonics, Bremen, Germany). Separation of metabolite standards was performed on a Supelco Ascentis Express C18-silica column (100 mm × 2.1 mm, 2.0 μm) (Sigma-Aldrich, Taufkirchen, Germany) with a gradient from 5 to 99.9% MeCN. Eluent A consisted of water with 0.1% (v/v) formic acid and eluent B of MeCN with 0.1% formic acid (v/v). Flow rate was set to 0.3 mL/min and column temperature was maintained at 40 °C. After 2 min of 5% B, %B was increased linearly to 99.9% within 15 min and held for 3 min. The column was re-equilibrated for 3 min. Aliquots of sample (5 μL) were injected via partial loop injection. Different methods for systematic evaluation of flow rate influences were derived from this standard method.

Metabolite standards were detected in positive and negative electrospray ionization mode with data-dependent acquisition of tandem MS. Source parameter were as followed: end plate offset = 500 V, capillary = 4500 V, nebulizer = 2.0 bar, dry gas = 10.0 L/min, dry temp = 200 °C, mass range = 50–1500.

#### LC–MS system 2

An Agilent 1200 HPLC equipped with a quaternary pump was coupled to a Bruker maXis plus UHR-ToF–MS. Separation of metabolite standards was performed on a Supelco Ascentis Express C18-silica column (150 mm × 2.1 mm, 3.0 μm) (Sigma-Aldrich, Taufkirchen, Germany) using the same solvents as on LC system 1. Flowrate was set to 0.4 mL/min. After 4.5 min of 5% A, %B increased linearly to 99.9% within 35.5 min and held for 6 min. MS parameters were similar to LC–MS system 1.

#### LC–MS system 3

LC–MS system 3 used the same column and gradient as LC–MS system 2, but the separation was performed on the hardware from LC–MS system 1 (Waters Acquity UPLC) differing in the gradient formation and delay volume. MS parameters were similar to LC–MS system 1.

### Setup of initial RI library

In total, 52 mixtures of up to 8 pooled standards were measured. The NAPS standards were injected always before and after each plate. Peaks were manually picked in Data Analysis 4.4 (Bruker Daltonics, Bremen, Germany) by creating extracted ion chromatograms. MS and MS/MS spectra were used for verification.

Calculation of RIs was performed in three different ways, either using the NAPS standards before or after the respective run or using the average of bracketing NAPS standards. RIs were individually calculated for each replicate run and then averaged. Functions for calculation of RIs have been implemented into the MetaCoreUtils package [18].

### Analysis and data processing of complex samples

*Caenorhabditis elegans* N2 and *Escherichia coli* NA22 were obtained from Caenorhabditis Genetics Center. Mixed-stage *C. elegans* were grown in liquid culture fed with *E. coli* NA22 and harvested by centrifugation. Metabolites were then extracted with 50% MeOH according to Witting et al. [19]. After extraction, solvent was evaporated and samples were re-dissolved in 20% MeCN to achieve an estimated concentration of roughly 10,000 worms/mL. For mouse plasma samples, proteins were precipitated by mixing 500 μL plasma with 1500 μL ice-cold MeCN, then vigorously vortexed and centrifuged for 15 min at 13,000 rpm at 4 °C. The supernatant was transferred to a fresh reaction tube and solvent evaporated. The sample was re-dissolved in 500 μL 20% MeCN. Aliquots of *C. elegans* metabolite and mouse plasma extracts and extracts spiked with NAPS at levels of 1:20, 1:40, and 1:80 were automatically processed with Genedata Expressionist for MS 13.5, which included *m*/*z* recalibration, noise reduction, RT alignment, peak picking, and isotope grouping. Results were exported as.xlsx file and further processed in Microsoft Excel 2016, and RI calculations were performed as described above. Matching of features on MS1 level using *m*/*z* or *m*/*z* and RI has been performed using the MetaboAnnotation package [20].

## Results and discussion

### Elution and MS(/MS) characteristics of NAPS

**Fig. 1:**
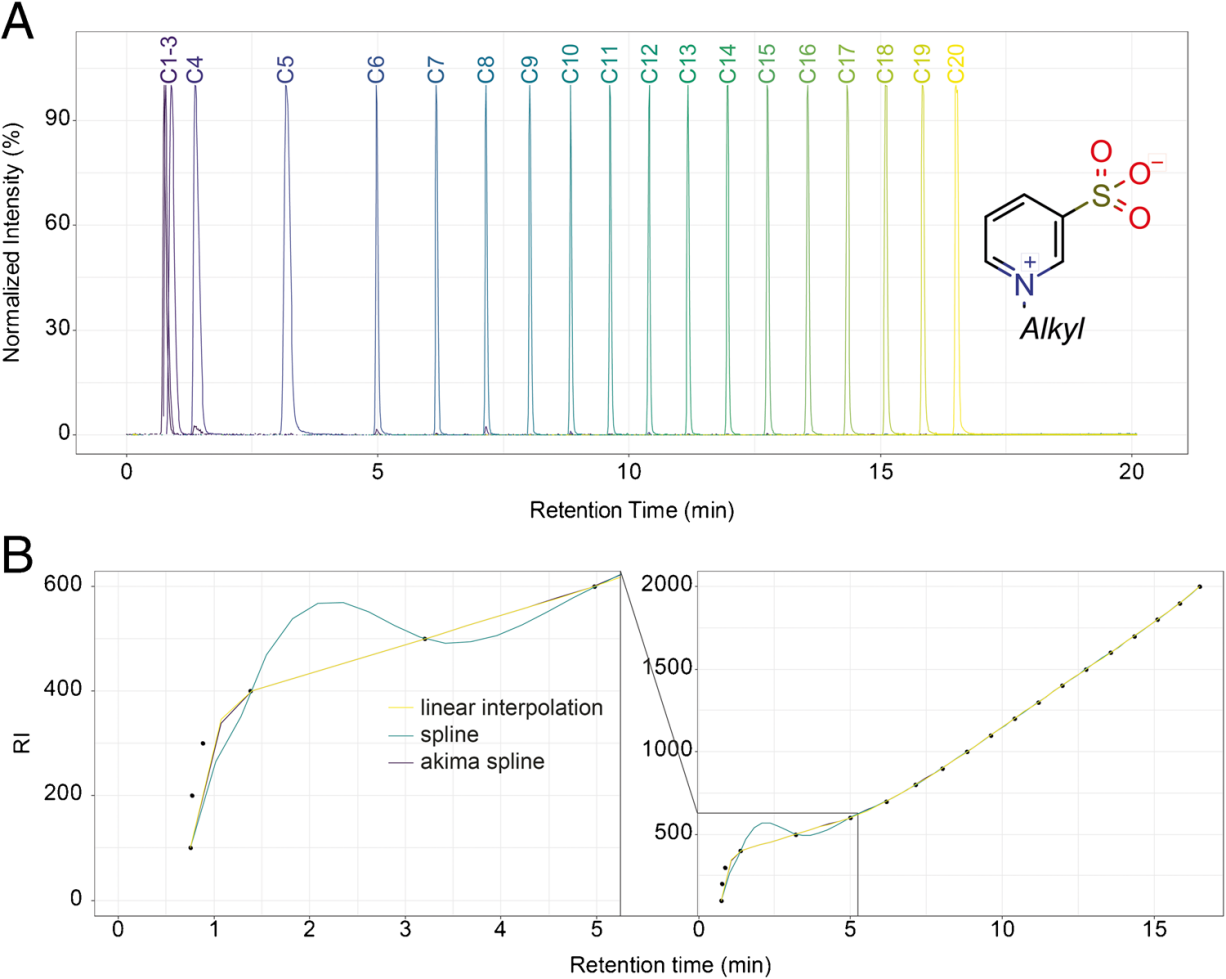
**A** Chromatogram showing the extracted ion chromatograms of all individual NAPS contained in the used mixture. The homologous series of NAPS shows a highly reproducible elution pattern with retention times increasing with chain length. The two permanent charges enable highly sensitive detection in ESI–MS and retention virtually independent of pH. Low retention of C1- to C3-NAPS is observed under the given analytical conditions. **B** Different fitting functions can be used to convert RTs to RI. For higher RT linear interpolation, cubic splines or Akima cubic splines lead to almost identical results. However, for lower RTs, cubic splines can overshoot due to strong changes in the gradient due to the requirement of steadiness at each nodal point. This is specific to the employed analysis conditions. Use of RP columns compatible with 100% aqueous eluents might increase retention of C1- to C3-NAPS and early eluting analytes, which should improve fitting

In order to index RTs, reference standards are required. *N*-Alkylpyridinium 3-sulfonates (NAPS) have been proposed by Quilliam as useful reference standards for indexing in reversed-phase LC–MS [15, 16]. Due to the two permanent and oppositely charged groups (quaternary imine and sulfonate), retention of NAPS is virtually independent of the separation pH. Furthermore, they can be detected in positive and negative ionization modes, as well with UV detectors due to the aromatic ring. In our employed chromatographic setup, NAPS ionize mainly as protonated ions, [M + H]^+^, in the positive mode and as formate adduct ions, [M + HCOO]^−^, in the negative ionization mode. Collision-induced fragmentation yields a common fragment of *m*/*z* 160.0063 in the positive mode ([C_5_H_6_NO_3_S]^+^) and *m*/*z* 79.9579 in the negative mode ([SO_3_]^−^), which makes the substances also useful for MRM or DIA-MS/MS workflows. At higher concentrations, we also observed gas-phase multimer formation (e.g., [2 M + H]^+^) as well as sodium adducts [M + Na]^+^ and sodiated multimers (e.g., [2 M + Na]^+^). The employed NAPS mixture (RM-RILC) consists of 20 homologues with length of the *N*-alkyl chain from 1 to 20. The retention index of the standards are denotated as 100 times the number of carbons in the alkyl chain (i.e., RI = 100 to 2000). The first three standards (C1-3, RI 100–300) elute in or close to the void volume (Fig. 1A and B) under our separation conditions and RTs of the C1-C3 analogs were nearly identical. Therefore, robust RIs could only be calculated for substances for which RTs were higher than the RT of the C3-NAPS standard. However, retention of C1 to C3 might be increased by the use of RP columns compatible with 100% aqueous eluents. Likewise, retention and potentially separation of early eluting metabolites might be improved.

### Setup of initial RI library

Initial experiments were all conducted on LC–MS system 1, which uses a high-pressure binary gradient pump. First, we constructed an RI library by measuring metabolite standards contained in the MSMLS using pools of rows, yielding mixtures not containing any isomeric or isobaric structures. After one initial injection of NAPS, always one plate was completely measured followed by one injection of NAPS so each sample was bracketed by two NAPS injections. Obtained retention times are available in SI Table 1. Out of 619 metabolite standards, 490 could be detected with our settings, 313 in both ion modes, 153 only in positive ion mode, and 24 only in negative ion mode. Chromatography was highly stable with maximum 6.1% and 5.4% relative standard deviation for RTs in the positive and negative modes. We further considered only standards eluting after C3-NAPS, reducing the number further to a total of 219 metabolites.

In the next step, we compared different ways of converting RTs to RIs. Functions for this conversion have been added to the MetaboCoreUtils R package. A generic function accepts as list of RTs for arbitrary substances as well as a matrix containing the index and RT of NAPS. The third argument is a function instructing how the conversion shall be performed. We tested three different functions, the first one performs linear interpolation according to Eq. 1, which is the default setting of the indexRtime() function in the MetaboCoreUtils package [18].

1 $$\mathrm{RI}={\mathrm{RI}}_{0}+({\mathrm{RI}}_{1}-{\mathrm{RI}}_{0})\frac{\mathrm{RT}-{\mathrm{RT}}_{0}}{{\mathrm{RT}}_{1}-{\mathrm{RT}}_{0}}$$

where RI_0_ and RI_1_ denominate the RI of the bracketing NAPS, which is the number of carbons in the alkyl chain multiplied by 100 and RT_0_ and RT_1_ the corresponding retention time. RT is the retention time of the substance for which the RI shall be calculated. Second the spline() function as well as aspline() function from the Akima package have been implemented [21]. The latter one is similar to the functionality used by Renaud et al. [16].

Each block of standard mixture injections was bracketed by two injections of NAPS standards, one before and one after the block. We compared different ways of calculating the RI. First, based on the NAPS run before, second on the NAPS injection run after, or lastly the average of bracketing runs was used as the reference for indexing. Each replicate was indexed individually to account for drifts in RT that might occur over time, e.g., based on column degradation and minor changes in solvent composition. In the first step, we used linear interpolation according to Eq. 1 for conversion of RT to RI and checked which difference is detected if the NAPS injection before or after the sample block or the average of both is used. The method of calculation had no significant influence and differences were in the range of the standard deviations of the individual calculations (deviations ranged from absolute values of 1 to 6 RI units, for metabolites with an RI > 300). Therefore, for all further work, we used the “average” method, which uses the average RT between the NAPS standard injected before and after each sample block, which might represent a good compromise also for larger sample batches.

In the next step, different other possibilities for conversion beside linear interpolation were compared. We have selected cubic splines or Akima cubic splines since they were suggested by Renaud et al. [16]. Comparing both against linear interpolation, we observed systematic differences for RI values < 750. Differences are very high for cubic splines and somehow lower for Akima cubic splines. These differences are based on the different fitting in the lower retention time region. Cubic splines tend to overshoot in regions with strong gradient changes due to the requirement steadiness at each nodal point. Generally, linear interpolation and Akima cubic splines tend to agree better. All R scripts are available from the SI.

### Systematic variation of flow rate

After construction of an initial RT/RI library and comparison of different ways for conversion of RT to RI, we checked if retention time indexing can compensate for different analytical settings. We systematically varied the flow rate of the used method and kept all other parameters constant. A binary HPG system was used for the construction of the library and four different flow rates (0.20, 0.25, 0.35, and 0.40 mL/min) have been additionally used. Since the elution order is known from the initial database construction, we measured larger pool samples with one pool per plate of the MSMLS and determined RTs in triplicates and performed individual conversion of each replicate using the average method with linear interpolation as described above. RTs from the different flow rates are available in SI Tables 2–5.

**Fig. 2:**
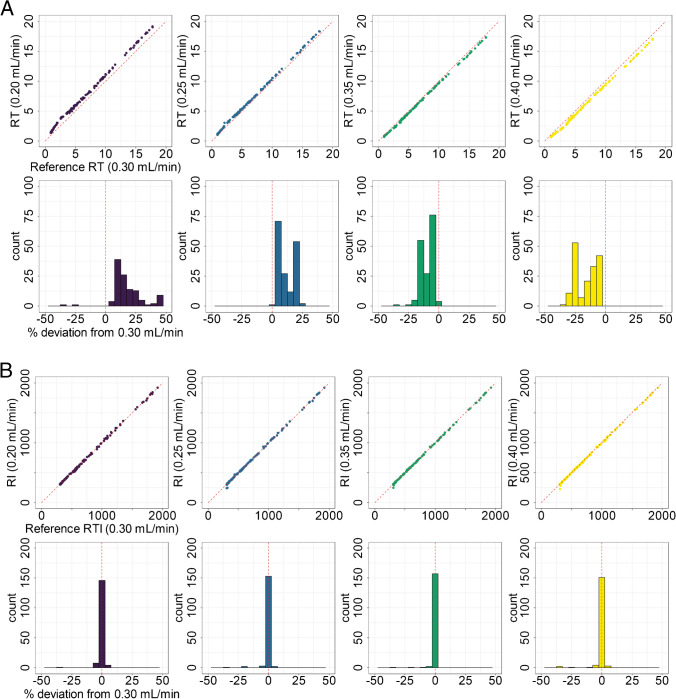
**A** Comparison of RTs between the reference method (0.30 mL/min) and other flow rates tested. For this comparison, all chromatographic parameters were kept constant, except for the flow rate. Systematic deviations of RTs under different flowrates compared to standard condition (0.30 mL/min) were observed. The upper row of plots shows the scatter plot of the RTs from each different flow rates plotted against the reference condition. The red dashed line indicates the diagonal, which represents a perfect fit. The lower row shows histograms of the relative deviation of RTs from the reference method in %. Flow rates lower than the reference flow rate showed higher RTs, while higher flow rates lead to decreased RTs. **B** Plots are similar to **A**, except that RI is used instead of RT. Good agreement between RIs from all conditions is found. All plots indicate that the conversion of RT to RI enables the normalization of the different flow rates

As expected, a systematic shift of RTs based on the flow rate was observed with lower flow rates showing a shift towards higher RTs, while higher flow rates resulted in faster elution (Fig. 2A). Differences in RT ranges from + 50 to − 30%, which demonstrates the difficulty of using RTs for cross-separation system comparison. RIs were calculated according to the Eq. 1 and relative deviations from the standard condition were calculated. By transforming RT to RI, the systematic trend is removed and makes the different conditions comparable. In the initial data, 18 substances showed deviations larger than 5% from the reference RI in one or more of the used conditions. We reinvestigated them and found that most of them were of very low abundance with low-quality MS1 and MS/MS data, which lead to wrong determination of RTs in initial peak picking. All other substances showed good agreement in their RIs. When plotting RIs from different conditions, slopes close to 1 and *R*^2^ > 0.9 were obtained.

### Cross-system comparison

In the next step, we wanted to test if conversion of RT to RI can be used to normalize retention data between different formats of separation systems. We chose a HPLC format column of the same column chemistry to test the comparability of RIs between HPLC and UHPLC. We employed a different LC–MS system (LC system 2) which was a HPLC system (max. pressure 400 bar) and showed generally higher system extra column volumes and is based on a different gradient mixing system (quaternary low-pressure gradient instead of binary high-pressure gradient) with a different gradient dwell volume and as well has a different particle size (3.0 μm instead of 2.0 μm). All substances have been measured in 7 pools, one for each plate similar to the experiment for varying the flowrates, and RTs and RIs were determined as for all other conditions. RTs are available in SI Table 6.

**Fig. 3:**
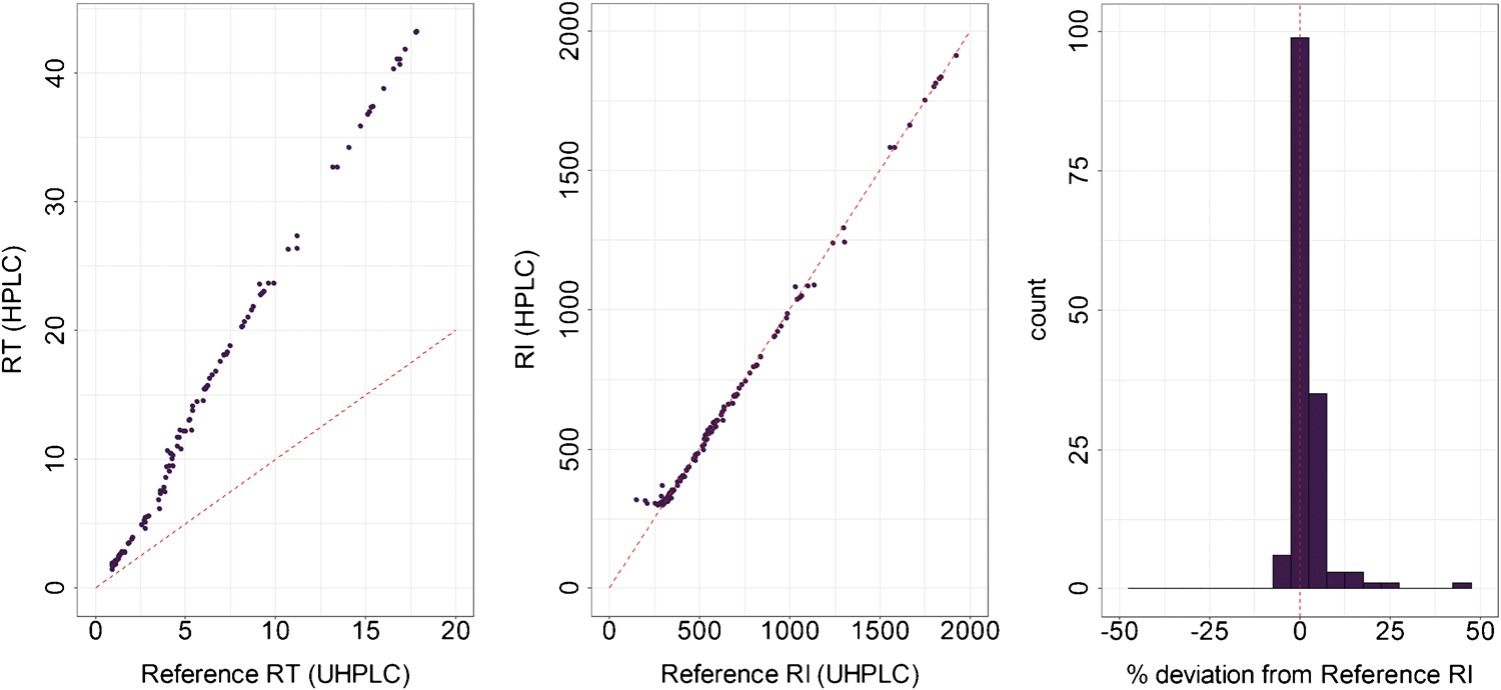
Conversion of RT to RI enables the normalization of retention data between HPLC and UHPLC format. Metabolite standards were measured on the identical phase system in HPLC format compared to the previously used UHPLC format. Analysis was performed on two different hardware setups differing in column dead volume, extra column dead volume, gradient dwell volume, and gradient mixing system (see “Material and methods”). While RTs (as expected) show a large deviation making a direct comparison not possible, RIs from the different systems are in good agreement. The red dashed lines indicate the diagonal, which represents a perfect fit. The histogram of the relative deviation of RIs from reference RIs in % shows no systematic deviation

As anticipated, RTs are highly correlated but are differing strongly between the UHPLC and HPLC system (Fig. 3A). However, normalization to RIs allowed the direct comparison of retention information from both systems, without the need of mapping functions. Regression of the RIs from the different LC systems showed a slope of 1.004 and a *R*^2^ of 0.998 (Fig. 3). The mean deviation between the two different systems using RIs was 0.25%. Seventy-four percent of all detected standards were between − 5.0 and + 5.0% indicating the high accuracy of the indexing method (Fig. 3C). RIs can therefore be used to normalize retention information and comparison of the different chromatographic systems.

### Application to biological matrices

#### Ion suppression effects of NAPS in biological samples

Next, we were interested in evaluating the performance of the NAPS RTI system with biological samples. Co-injection of NAPS together with the biological matrix of interest represents the most accurate application for RTI, since any run-to-run deviations in RTs due to variations of conditions or matrix effects will be eliminated. In order to evaluate if RIs can be used with biological matrices, we used the HPLC column from LC system 2 (150 × 3 mm, 3 μm) in LC system 1 (UHPLC) to create a third separation system (LC–MS system 3), which is different from the previous ones and therefore represents an independent validation. *C. elegans* and mouse plasma metabolite extracts served as biological matrices.

First, we evaluated the potential matrix effect on the RTs of individual NAPS. NAPS solution was either diluted with one of the biological two matrices or with 20% MeCN at dilution factors of 1:20, 1:40, and 1:80 and injected to the LC–MS. No significant shift in RTs of the NAPS between injection in pure solvent or in matrix was observed (data not shown). Second, we investigated the different ion species of NAPS detectable in biological matrices. At higher concentrations, NAPS form different adducts in the gas-phase including different multimers. Since they are covering a large RT and *m*/*z* range, they might be useful for recalibration in future HRMS approaches. We therefore checked also the dependency of adducts and multimers on the dilution factor with samples. In the positive mode, we included the following adducts: [M + H]^+^, [2 M + H]^+^, [3 M + H]^+^, [M + Na]^+^, [2 M + Na]^+^, and [3 M + Na]^+^. In the negative ion mode, we used [M − H]^−^, [2 M − H]^−^, [3 M − H]^−^, [M + HCOO]^−^, [2 M + HCOO]^−^, and [3 M + HCOO]^−^. At a dilution of 1:20, most of the tested adducts in the positive and negative ion modes could be detected, with the exception of NAPS 100 to 300 for which only the [M + H]^+^ or [M + HCOO]^−^ could be detected. [3 M − H]^−^ adducts were not detected and [2 M − H]^−^ only for middle and long chains (C8-20). 

**Fig. 4:**
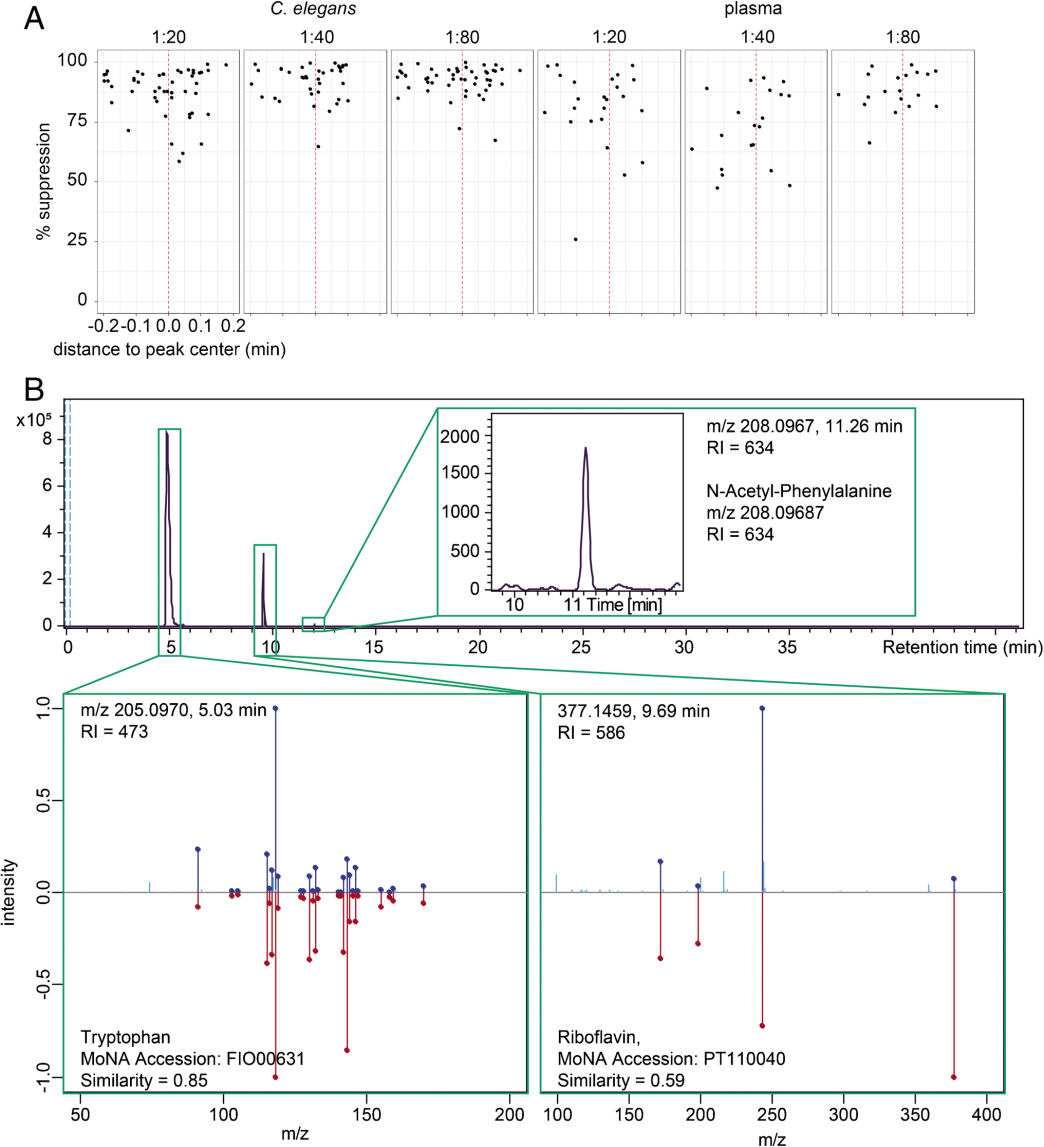
**A** Ion suppression effects of NAPS. The NAPS mixture was spiked into *C. elegans* or plasma metabolite extracts at different dilution factors and ion suppression was evaluated by comparison against unspiked matrix. The effect of suppression was checked in relation to the distance of a metabolite feature to the apex of the closest NAPS standard. The example shows the % suppression between − 0.2 and 0.2 min from the apex of the C15-NAPS. **B** Selection of annotation results from positive ionization mode of detected *C. elegans* metabolites measured using LC–MS system 3 (see “Material and methods”). Annotation was performed in two steps. First matching was performed on *m*/*z* and RI using the MetaboAnnotation package within R. In a second step, acquired MS^2^ spectra have been compared with library spectra from MoNA of the respective matching metabolite standard. N-Acetyl-phenylalanine was only matched based on *m*/*z* and RI values. Data for the different features and their respective RI values on the different LC–MS systems can be found in Table 1

Third, since NAPS have two permanent charges, they potentially cause ion suppression for co-eluting metabolites. We evaluated ion suppression by comparing intensities of metabolites eluting in the time range of the individual NAPS. The first three NAPS standards (100–300) are eluting together with many polar metabolites in or near the void volume, where high suppression is usually observed. Therefore, only NAPS standards with RI > 300 were evaluated. Peaks eluting in the range of ± 0.20 min around the RT of the respective NAPS were evaluated, and their maximum intensities were compared against non-spiked matrix. Internal *m*/*z* recalibration, chromatographic alignment, peak peaking, and isotope clustering have been performed in Genedata Expressionist for MS 13.5 and the maximum intensity for each isotope cluster was exported. Relative values compared to non-spiked matrix were calculated: 100% would indicate no ion suppression, values < 100% ion suppression and values > 100% ion enhancement. NAPS chromatographic peaks generally span a width of about 0.2 min. To investigate the effect of ion suppression, we plotted the RT distance of a feature to the RT of the respective NAPS. With increasing dilution factor, ion suppression is reduced, as shown in Fig. 4 for the C15 NAPS in the positive ionization mode. For both investigated matrices, higher dilutions lead to reduced ion suppression, although 100% is never fully achieved (Fig. 4B). Similar trends were seen in the negative ionization mode (data not shown). However, these effects must be evaluated carefully for each matrix and LC–MS system. If suppression effects might be still too strong, a separate batch of QC samples could be spiked with NAPS to determine RTs in matrix but not directly affect the biological and QC samples of the metabolomics studies. This spiked QC could be injected after each QC sample every ten samples, which is sufficiently stable to perform RTI.

**Table 1:** Results of metabolite annotation of features detected in *C. elegans* using the constructed RI database. *C. elegans* metabolite extracts were measured on the LC–MS system 3 described in the “Material and methods” section. RT and RI values from LC–MS systems 1 and 2 were used. LC–MS systems 2 and 3 used the identical column, but different hardware leading to differences in system extra column volume, mixing system, and gradient dwell volume. Conversion of RT to RI allowed the use of retention information for metabolite annotation. Values in brackets indicate the relative error

Ion mode	Feature (system 3)	Annotation	Type	UPLC (system 1)	HPLC (system 2)
Positive	*m*/*z* 205.0970 RT 5.03 min RI 472	Tryptophan [M + H]^+^	RT	2.71 (46%)	5.29 (5%)
			RI	474 (0.4%)	475 (0.6%)
	*m*/*z* 377.1459 RT 9.69 min RI 586	Riboflavin [M + H]^+^	RT	4.66 (52%)	12.31 (27%)
			RI	582 (0.7%)	595 (1.5%)
	*m*/*z* 208.0967 RT 11.26 min RI 634	*N*-Acetylphenylalanine [M + H]^+^	RT	5.38 (52%)	13.83 (23%)
			RI	633 (0.2%)	643 (1.4%)
	*m*/*z* 139.0389 RT 6.51 min RI 513	4-Hydroxy benzoic acid [M + H]^+^	RT	3.4 (48%)	6.85 (5.2%)
			RI	511 (0.4%)	521 (1.6%)
		3-Hydroxy benzoic acid [M + H]^+^	RT	4.30 (34%)	9.54 (46%)
			RI	561 (9.4%)	564 (9.9%)
		Salicylic acid [M + H]^+^	RT	4.30 (34%)	9.54 (46%)
			RI	561 (9.4%)	564 (9.9%)
Negative	*m*/*z* 137.0250 RT 6.53 min RI 513	4-Hydroxybenzoic acid [M − H]^−^	RT	3.4 (48%)	6.85 (4.9%)
			RI	511 (0.4%)	521 (1.6%)
		3-Hydroxybenzoic acid [M − H]^−^	RT	4.30 (34%)	9.54 (46%)
			RI	561 (9.4%)	564 (9.9%)
		Salicylic acid [M − H]^−^	RT	4.30 (34%)	9.54 (46%)
			RI	561 (9.4%)	564 (9.9%)
	*m*/*z* 375.1306 RT 9.69 min RI 586	Riboflavin [M − H]^−^	RT	4.65 (52%)	12.31 (27%)
			RI	582 (0.7%)	595 (1.5%)

#### Use of RIs for metabolite annotation

Finally, we tested if RIs can be used for annotation of metabolites using similar chromatographic setups but differing in the instrumentation. We used the data obtained from the biological matrices on the 3 μm column in the UPLC system (LC system 3). Compared to the measurements on LC system 2, NAPS showed RT differences of up to 3 min, which is due to reduced system extra column volume and differences in the formation of the gradient (HPG vs LPG pump) and differences in gradient dwell volume.

We used the NAPS co-injected with the samples to convert RTs to RIs and use *m*/*z* values for initial annotation. We performed annotation of features detected in *C. elegans* with our initial database obtained on the UHPLC-UHR-ToF–MS system (LC system 1) as well as with the database from the HPLC–MS system (LC system 2). Annotation was performed using the matchMz() function from the MetaboAnnotation package either only using *m*/*z* or *m*/*z* and RI [20].

Using only *m*/*z* values with an error of 0.005 Da, 108 features were putatively annotated with one or several metabolites from the database in the positive ionization mode. This number is reduced to 40 if additionally the RI is used with a maximum error of 10 RI units. We closely examined some of these features. First, a peak at the 5.03 min with *m*/*z* 205.0970 was annotated as the [M + H]^+^ adduct of tryptophan. The RIs in our initial databases are 475 and 474 respectively and the RI in the sample was 472 which reflects an error of 2 RI units or 0.4%. When comparing the RT obtained on the same column with the same gradient on the Agilent 1200 HPLC system, a difference of 5% was observed, while for the RT used in the initial DB construction using a Waters Acquity UPLC, a difference of 46% was found. This peak has been selected for fragmentation using data-dependent acquisition of MS/MS, and we therefore compared also obtained MS^2^ spectrum. Spectra from the standard found in the MSMLS and the peak detected in *C. elegans* showed a perfect match (data not shown). An additional spectral similarity search was performed on the MassBank of North America (MoNA). The closest hit was indeed tryptophan measured on a similar instrument (MoNA Accession FIO00631). Cosine similarity was 0.85. The second example was a feature with *m*/*z* 377.1459 detected at 9.69 min annotated as [M + H]^+^ of riboflavin. Likewise, we checked MS/MS data that was available and were able to match a fragmentation spectrum of riboflavin (MoNA Accession PT110040). Lastly, *m*/*z* 208.0967 at 11.26 min was annotated as *N*-acetylphenylalanine. This peak was low in intensity and no MS/MS collection was triggered. Beside *m*/*z* value, RI was used for annotation, which increased the confidence of the identification. Results from the identification are shown in Table 1 and Fig. 4B.

So far, all examples had only one annotation based on the database. However, RI shall be used in future as orthogonal information for metabolite identification, which allows the filtering out of false-positive annotations from *m*/*z* values alone. We therefore checked for detected features that showed multiple annotations based on *m*/*z* alone. A signal with *m*/*z* 139.0389 detected at 6.51 min was annotated as [M + H]^+^ of either 3-hydroxybenzoic acid, 4-hydroxybenzoic acid, or salicylic acid (2-hydroxybenzoic acid). All three are isomers with the same sum formula C_7_H_6_O_3_ and very similar fragmentation pattern only differing in the abundance of different fragments. Therefore, differences in retention behavior are required to correctly identify the identity. Using additionally the RI, results were reduced to a single hit, 4-hydroxbenzoic acid. 4-Hydroxybenzoic acid known to be present in *C. elegans* is one of the building blocks of complex ascaroside signaling molecules [22]. Identifications of 4-hydroxybenzoic acid and riboflavin could be confirmed also in the negative ionization mode using matching *m*/*z* and RI values.

## Conclusion

Retention information is important for the identification of metabolites, since it is orthogonal to MS and MS/MS. We presented a way to normalize retention information in reversed-phase-LC–MS-based metabolomics employing a mixture of homologues NAPS to convert RTs to RI. We compared different indexing strategies using different fitting functions. Based on authentic chemical standards, we compared differences in flow rates and have shown that conversion of RTs to RIs allows normalization for different experimental settings. Furthermore, separations carried on different instruments (UPLC vs HPLC) can be compared. This comparison showed very low deviations in the RIs making it possible in the future to compare retention data from different systems using the same separation chemistry (the same column and eluent chemistry). NAPS can be co-injected with the sample for most accurate RTI, but ion suppression might be an issue. Our results indicate that at a sufficient dilution, NAPS are still detectable, but ion suppression effects are reduced. However, the dilution factor must be tested and optimized for each matrix and mass spectrometric setup individually for best performance. Lastly, we have shown that RIs can be used to improve the annotation and identification of metabolites by adding an additional orthogonal parameter to *m*/*z* and fragmentation pattern. Our results have shown that isomeric species can filter RIs obtained on a different LC–MS setup. The current database is of limited size, and in the future, it has to be populated with additional data. It might be risky to use *m*/*z* and RI values alone for metabolite identification. Instead, matching of RIs shall be used to re-rank results from tandem MS search or combined with accurate mass and tandem MS matching in an integrated (consensus) scoring function. Furthermore, we would like to point out that in our case especially for early eluting metabolites, it is important to use the same indexing functions as used in the initial database construction to avoid systematic differences.

Based on our findings, we conclude that RTI in RP-LC–MS will be a useful tool towards standardized reporting as well as re-use of chromatographic data. In contrast to GC–MS, it remains elusive how well RIs generated with different mobile and stationary phases can be compared with each other. First, experiments indicate that changes are only minor between comparable C18-silica stationary phases. Likewise, a similar indexing system is missing for HILIC-based separations.

## Supplementary Information

Below is the link to the electronic supplementary material.

Supplementary file1 (RAR 4 KB) SI Scripts: All R scripts required for conversion of RTs into RIs.

Supplementary file2 (XLSX 245 KB) SI Table 1: Retention times of all standards obtained from the UHPLC-MS system (System 1)

Supplementary file3 (XLSX 246 KB) Si Table 2-4: Retention times of all standards obtained from UHPLC-MS (System 1) with varying flow rates

Supplementary file4 (XLSX 245 KB)

Supplementary file5 (XLSX 244 KB)

Supplementary file6 (XLSX 244 KB)

Supplementary file7 (XLSX 243 KB) SI Table 6: Retention times of all standards obtained from the HPLC-MS system (System 2)

## References

[1] Aicheler F, Li J, Hoene M, Lehmann R, Xu G, Kohlbacher O (2015). Retention time prediction improves identification in nontargeted lipidomics approaches. Anal Chem.

[2] Sinclair E, Hollywood KA, Yan C, Blankley R, Breitling R, Barran P (2018). Mobilising ion mobility mass spectrometry for metabolomics. Analyst.

[3] Sumner L, Amberg A, Barrett D, Beale M, Beger R, Daykin C (2007). Proposed minimum reporting standards for chemical analysis. Metabolomics.

[4] Witting M, Böcker S (2020). Current status of retention time prediction in metabolite identification. J Sep Sci.

[5] Kováts E (1958). Gas-chromatographische Charakterisierung organischer Verbindungen. Teil 1: Retentionsindices aliphatischer Halogenide, Alkohole, Aldehyde und Ketone. HCA.

[6] Strehmel N, Hummel J, Erban A, Strassburg K, Kopka J (2008). Retention index thresholds for compound matching in GC–MS metabolite profiling. J Chromatogr B Analyt Technol Biomed Life Sci.

[7] Aderjan R, Bogusz M (1988). Nitroalkanes as a multidetector retention index scale for drug identification in gas chromatography. J Chromatogr A.

[8] Smith RM. Chapter 3 Retention index scales used in high-performance liquid chromatography. In: Smith RM, editor. J Chromatogr Library. 57: Elsevier; 1995. p. 93–144.

[9] Hall LM, Hall LH, Kertesz TM, Hill DW, Sharp TR, Oblak EZ (2012). Development of Ecom(50) and retention index models for non-targeted metabolomics: identification of 1,3-dicyclohexylurea in human serum by HPLC/mass spectrometry. J Chem Inf Model.

[10] Allen F, Greiner R, Wishart D (2015). Competitive fragmentation modeling of ESI-MS/MS spectra for putative metabolite identification. Metabolomics.

[11] Dührkop K, Shen H, Meusel M, Rousu J, Böcker S (2015). Searching molecular structure databases with tandem mass spectra using CSI:FingerID. Proc Natl Acad Sci.

[12] Wolf S, Schmidt S, Müller-Hannemann M, Neumann S (2010). In silico fragmentation for computer assisted identification of metabolite mass spectra. BMC Bioinformatics.

[13] Samaraweera MA, Hall LM, Hill DW, Grant DF (2018). Evaluation of an artificial neural network retention index model for chemical structure identification in nontargeted metabolomics. Anal Chem.

[14] Zheng S-J, Liu S-J, Zhu Q-F, Guo N, Wang Y-L, Yuan B-F (2018). Establishment of liquid chromatography retention index based on chemical labeling for metabolomic analysis. Anal Chem.

[15] Quilliam MA. Retention index standards for liquid chromatography, Patents US 2017/0102367 A (2017) and US 10,228,356 B2 (2019).

[16] Renaud JB, Hoogstra S, Quilliam MA, Sumarah MW (2021). Normalization of LC-MS mycotoxin determination using the N-alkylpyridinium-3-sulfonates (NAPS) retention index system. J Chromatogr A.

[17] Rigano F, Arigò A, Oteri M, La Tella R, Dugo P, Mondello L (2021). The retention index approach in liquid chromatography: an historical review and recent advances. J Chromatogr A.

[18] Rainer J, Witting M. MetaboCoreUtils: Core Utils for Metabolomics Data. R package version 1.0.0 2021 [Available from: https://bioconductor.org/packages/release/bioc/html/MetaboCoreUtils.html. Accessed 20 Nov 2021.

[19] Witting M, Lucio M, Tziotis D, Wägele B, Suhre K, Voulhoux R (2015). DI-ICR-FT-MS-based high-throughput deep metabotyping: a case study of the Caenorhabditis elegans–Pseudomonas aeruginosa infection model. Anal Bioanal Chem.

[20] Rainer J, Vicini A, Witting M. MetaboAnnotation 2021 [Available from: https://github.com/rformassspectrometry/MetaboAnnotation. Accessed 20 Nov 2021.

[21] Akima H, Gebhardt A, Petzold T, Maechler M. akima: interpolation of irregularly and regularly spaced data. 2021. Available from https://cran.r-project.org/web/packages/akima/index.html.

[22] von Reuss SH, Bose N, Srinivasan J, Yim JJ, Judkins JC, Sternberg PW (2012). Comparative metabolomics reveals biogenesis of Ascarosides, a modular library of small-molecule signals in C. elegans. J Am Chem Soc..

